# Life's essential 8 and risk of all‐cause mortality in individuals with cardiovascular diseases: A prospective community‐based study

**DOI:** 10.1002/clc.24119

**Published:** 2023-11-23

**Authors:** Lili Huang, Aitian Wang, Zhijun Wu, Shuohua Chen, Yan Zheng, Shouling Wu, Xiang Gao

**Affiliations:** ^1^ Department of Nutrition and Food Hygiene School of Public Health, Institute of Nutrition Shanghai People's Republic of China; ^2^ Department of Intensive Medicine Kailuan General Hospital Tangshan People's Republic of China; ^3^ Department of Cardiovascular Medicine, Ruijin Hospital Shanghai Jiaotong University School of Medicine Shanghai People's Republic of China; ^4^ Department of Cardiology Kailuan General Hospital Tangshan People's Republic of China; ^5^ State Key Laboratory of Genetic Engineering, Human Phenome Institute, and School of Life Sciences Fudan University Shanghai People's Republic of China

**Keywords:** all‐cause mortality, cardiovascular disease, cardiovascular health, prevention

## Abstract

**Background:**

Although risk factors for mortality in individuals with cardiovascular diseases (CVD) have been reported, little is known regarding the association between the comprehensive cardiovascular health (CVH) index assessed by life's essential 8 (LE8) and the risk of mortality.

**Hypothesis:**

The aim of this study was to evaluate the CVH assessed by LE8 and risk of mortality in individuals with CVD.

**Methods:**

A total of 1391 participants with CVD diagnosed before 2014 from the Kailuan cohort were included in the analysis. The CVH score ranged from 0 to 100 was assessed using the LE8 metrics (diet quality, physical activity, sleep health, cigarette smoking, body mass index, lipids, blood glucose, and blood pressure). Cox regression model was used to estimate the association between the CVH score and risk of all‐cause mortality.

**Results:**

During a mean follow‐up of 6.1 ± 1.5 years, 229 deaths occurred. The hazard ratio for all‐cause mortality was 0.57 (95% confidence interval [CI]: 0.38, 0.84) in the highest quartiles compared with the lowest quartiles of CVH scores and 0.85 (95% CI: 0.75, 0.95) for each 10 points increment in CVH scores (*p*_trend_ = .009), after adjustment for age, sex, CVD duration, social‐economic status, alcohol consumption, inflammation, medicine use, and kidney function. We did not observe significant interactions between the CVH scores and age, sex, and duration of CVD diagnosis (*p*_interaction_ > .05 for all).

**Conclusions:**

The CVH assessed by the LE8 metrics was associated with a lower risk of all‐cause mortality in individuals with CVD.

## Graphical abstract

In this cohort study of 1391 participants with cardiovascular diseases from the Kailuan cohort in China, the cardiovascular health (CVH) was assessed by life's essential 8. Participants with the highest CVH scores compared with the lowest CVH scores had nearly 50% lower risk for all‐cause mortality.

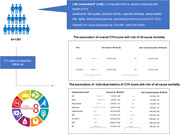

## 1 INTRODUCTION

Cardiovascular diseases (CVDs), including ischemic heart disease and stroke, remain the leading cause of death globally, although clinical treatments have been improved significantly. The morbidity and mortality of CVD have been increasing during the past three decades^1^; thus, there is an urgent need to target modifiable factors to mitigate this major public health problem. To date, the factors associated with mortality in individuals with CVD were mainly focused on single factors, such as diet quality,^2^ physical activity (PA),^3^ smoking,^4^ and lipid control.^5^ There is a paucity of data examining the overall effect of modified factors, including lifestyle and metabolic factors, on mortality risk in individuals with CVD.

Recently, the American Heart Association proposed a composite index to assess cardiovascular health (CVH), called “life's essential 8” (LE8), including four behavioral factors (diet quality, PA, cigarette smoking, and sleep health) and four metabolic factors (body mass index [BMI], lipids, fasting blood glucose (FBG), and blood pressure [BP]).^6^ A study quantified CVH levels in the US population using this index and observed significant differences in age, sex, and racial group.^7^ The original purpose of this CVH index was for the primary prevention of CVD risk. However, given the impact of single metrics of LE8 on the prognosis of CVD in previous studies, it is necessary to examine the effect of overall CVH assessed by LE8 on the prognosis of CVD, such as mortality. In this context, we prospectively investigated the association between CVH scores and all‐cause mortality among individuals with CVD in two ongoing Chinese cohorts.

## 2 METHODS

### 2.1 Study population

The participants included in this study were participants of the Kailuan study I, a large population‐based cohort carried out from 2006 to 2007, including 101 510 Chinese adults, and the Kailuan study Ⅱ, which was started from 2008 to 2010, recruiting 35 856 adults. The detailed study design was described previously.^8^, ^9^ Briefly, participants in the two cohort studies were required to complete questionnaires and clinical and laboratory examinations. They then were followed biennially with the same strategies in 11 hospitals affiliated with the Kailuan Group. In 2014, a validated food‐frequency questionnaire and PA questionnaire were administered. We thus treated 2014 as the baseline for the current analysis.

A total of 4024 (3654 in Kailuan Ⅰ study and 370 in Kailuan Ⅱ study) participants were diagnosed with CVD (myocardial infarction [MI], ischemic stroke, and hemorrhagic stroke) based on medical records in or before 2014. We excluded those with cancer (*n* = 82) at baseline and without completed CVH data (*n* = 2551), resulting in 1391 participants for analysis (1112 in Kailuan Ⅰ study and 279 in Kailuan Ⅱ study) (Supporting Information: Figure S1). Compared with participants included in the final analysis, those excluded tended to be older; were more likely to be coal miners; had higher levels of income per month and high‐sensitivity C‐reactive protein (hs_CRP), and had a higher proportion of lowering BP medication use (Supporting Information: Table S1).

The present study was approved by the Ethics Committee of the Kailuan Medical Group. All participants signed written informed consent at the survey enrollment.

### 2.2 CVH measurements

CVH was assessed by LE8, including four lifestyle and four metabolic factors.

#### 2.2.1 Lifestyle factors

Dietary intake was collected via a validated food frequency questionnaire which has been used in other studies among Chinese populations.^10^, ^11^ Diet quality was assessed via the healthy eating index (HEI)‐2015 scores^12^ and the dietary approaches to stop hypertension (DASH) scores^13^ as described in previous studies. The total HEI‐2015 scores ranged from 0 (worst) to 100 (best) points,^12^ and total DASH scores ranged from 9 (worst) to 45 (best) points.^13^ PA was assessed by a validated international PA questionnaire, and the score was calculated based on the minutes of moderate‐ (to vigorous) intensity activity per week.^6^ Sleep health was measured via self‐reported questionnaires,^14^ and sleep duration per night was used to calculate CVH scores. The information on cigarette smoking was also collected using questionnaires and then categorized into five groups: “never,” “former, quit≥5 years,” “former, quit between 1 and 5 years,” “former, quit< 1 year,” or “current.”

#### 2.2.2 Metabolic indicators

Weight and height were measured by trained staff using standardized procedures.^15^ BMI was calculated as weight in kilograms divided by height in meters squared.

Fasting (8‐ to 12‐h) blood samples were collected in the morning and then stored at −80°C at the central laboratory of Kailuan general hospital. The methods that measured blood lipids and FBG were described previously.^16^, ^17^ The blood total cholesterol and triglyceride concentrations were determined using the endpoint test and enzymatic colorimetric methods, respectively (Mind Bioengineering Co. Ltd). The high‐density lipoprotein cholesterol (HDL‐C) and low‐density lipoprotein cholesterol concentrations were measured with the direct test method (Mind Bioengineering Co. Ltd). The inter‐ and intracoefficient of variations for each measurement was <10%. Non‐HDL‐C as a metric of LE8 was calculated by subtracting HDL‐C concentration from total cholesterol.

FBG concentrations were measured using the hexokinase/glucose‐6‐phosphate dehydrogenase method. The coefficient of variation using blind quality control specimens was <2.0%.

BP was measured using a mercury sphygmomanometer according to the standard recommended procedures.^18^ All participants received at least 2 measurements of BP after at least a 5‐minute rest. The average value of the multiple BP measures was used for further analysis.^19^

The information on lipid‐lowering, glucose‐lowering, and antihypertensive medication use was obtained via questionnaires.

#### 2.2.3 Quantification of CVH scores

The detailed calculation of CVH scores based on LE8 was described by the American Heart Association Presidential Advisory guideline.^6^ The value of each metric of LE8 was assigned piecewise, ranging from 0 to 100. Because the participants of this study were individuals with CVD, we did not subtract 20‐point scores for individuals who used lipid‐lowering or antihypertensive medications (however, results after subtracting the 20‐points for the use of medications did not materially change, data not shown). The overall CVH score was calculated by summing the eight individual scores and then dividing by eight. As two dietary patterns (HEI‐2015 and DASH) were available, in this study, we used the CVH score based on the HEI‐2015 as the primary exposure (referred to as CVH‐HEI in this manuscript). We also reported results for CVH score based on the DASH (referred to as CVH‐DASH).

### 2.3 Outcome ascertainment

The primary outcome of this study was all‐cause mortality. Detailed death determination methods were described previously.^20^ The information on fatal events was collected through a review of death certificates from provincial vital statistics offices, hospital records, medical insurance data, and next of kin, relatives, or eyewitnesses where was possible.

### 2.4 Covariate measurements

Data on age, sex, family history of CVD, duration of CVD diagnosis, education, average income per month, occupation, and medication use were obtained from a standardized questionnaire at baseline and then updated biennially. Alcohol consumption was assessed via the food frequency questionnaire at baseline. Waist circumference was measured with a nonstretchable tape by trained staff. Serum creatinine was measured using the sarcosine oxidase assay method (BioSino Bio‐technology and Science Inc.) and combined with age and sex to calculate the estimated glomerular filtration rate (eGFR) according to the Chronic Kidney Disease Epidemiology Collaboration equation.^21^ Hs‐CRP concentrations were measured using a high‐sensitivity particle‐enhanced immunonephelometry assay (Cias Latex CRP‐H, Kanto Chemical Co. Inc.).^22^

### 2.5 Statistical analysis

The participants' characteristics across CVH score quartiles were compared, with one‐way analysis of variance, Kruskal–Wallis test, or chi‐square tests being applied where appropriate. The person‐time of follow‐up was calculated from the date of the 2014 survey to either the date of death or the end of follow‐up (December 31, 2021). Cox regression was used to examine the association between CVH scores and the risk of all‐cause mortality. CVH scores were treated as quartiles, and the lowest quartile was considered as the reference. The linear trend in risk across CVH quartiles was tested by using the median value for each quartile and treating them as continuous variables. The inclusion of covariates was mainly based on previously published literature.^2^, ^23^, ^24^, ^25^ In this study, we adjusted for age (continuous), sex (men or women), occupation (coal miners, other blue collars, or white collars), education (illiterate and primary, middle school or high school and above), average income per month (≤1000, 1001–3000, >3000 RMB, or missing), alcohol consumption (never and past, current or missing), hs‐CRP (≤3 or >3 mg/L), waist circumference (quartiles), eGFR (quartiles), duration of CVD diagnosis (quartiles), family history of MI and stroke (yes or no), glucose‐lowering medications (yes or no), lipid‐lowing medications (yes or no), and antihypertensive medications (yes or no).

Further, we analyzed the association of the CVH scores with all‐cause mortality risk stratified by CVD types (MI or stroke) and the association of each individual metric of LE8 with all‐cause mortality risk.

Interactions of CVH with age, sex, and duration of CVD diagnosis were examined using a likelihood ratio test, adjusting for aforementioned covariates, and subgroup analyses were performed when the interaction term was significant.

Several sensitive analyses were conducted. Considering the possibility of reverse causality, we excluded those who died in the first 2 years of follow‐up (*n* = 44). In addition, we excluded coal miners (*n* = 292), considering the generalizability of the results.

The statistical analyses in this study were performed by R (version 3.6.2); *p* < .05 (two‐sided) was considered statistically significant.

## 3 RESULTS

### 3.1 Cohort characteristics

In this study population, the mean age at baseline was 62.6 years old, 89.2% were men, and the mean duration of CVD diagnosed were 3.85 years (Supporting Information: Table S1). Participants with higher CVH‐HEI scores were older, more likely to be women, and had low levels of income per month and eGFR relative to those with lower scores (Table 1).

**Table 1:** Baseline characteristics of individuals with CVDs in the Kailuan study across CVH with HEI‐2015 quartiles.

	CVH‐HEI				
	Quartile1	Quartile2	Quartile3	Quartile4	*p*_trend_
N	410	436	433	452	
Age, year	60.8 ± 8.61	62.3 ± 9.02	64.1 ± 9.09	63.2 ± 10.1	<.001
Men, %	92.8	90.3	87.5	86.5	.03
Family history of CVD, %	12.9	13.4	12.8	11.0	.78
Lipid‐lowering medications, %	5.1	4.6	4.3	3.3	.69
Antihypertensive medications, %	37.2	38.3	40.0	30.3	.04
Glucose‐lowering medications, %	18.3	13.1	7.8	3.0	<.001
Education level, %					
Illiterate and primary	6.3	7.7	7.5	9.6	.42
Middle school	76.9	79.4	79.4	74.7	
High school and above	16.8	12.9	13.0	15.7	
Occupation, %					
Coal miners	23.1	20.6	17.7	22.6	.28
Other blue collars	66.4	70.9	67.5	65.0	
White collars	8.7	6.6	11.3	9.4	
Missing	1.8	2.0	3.5	3.0	
Average income, RMB/month, %					
≤1000	34.8	43.4	42.0	47.1	.005
1001–3000	55.9	50.3	48.1	40.8	
>3000	8.4	6.0	8.1	10.2	
Missing	0.2	0.1	0.4	0.5	
Alcohol consumption, %					.07
Never	66.1	76.9	75.4	79.9	
Current, light	21.9	14.0	14.2	12.9	
Current, moderate	4.5	3.7	3.2	1.9	
Current, heavy	0.9	0.6	0.3	0.3	
Current, unknown	0	0.3	0.3	0.3	
Past	0.9	0.6	0.0	0	
Missing	5.7	4.0	5.8	4.7	
Hs_CRP > 3 mg/L, %	23.4	23.7	21.4	17.6	.17
Duration of CVD, year	3.81 ± 2.35	3.76 ± 2.35	3.88 ± 2.40	3.95 ± 2.30	.33
WC, cm	91.7 ± 9.95	89.7 ± 9.47	88.4 ± 9.01	87.3 ± 8.16	<.001
eGFR, ml/min/1.73m^2^	92.0 ± 21.0	92.2 ± 19.8	88.0 ± 20.8	89.6 ± 19.5	.02

Abbreviations: CVD: cardiovascular disease; CVH: cardiovascular health; eGFR: estimated glomerular filtration rate; HEI, healthy eating index; Hs_CRP: high‐sensitivity C‐reactive protein; WC: waist circumference.

### 3.2 CVH scores and risk of all‐cause mortality

During a mean follow‐up period of 6.1 ± 1.5 years, 229 death cases were identified. The age‐adjusted hazard ratio (HR) for the highest versus lowest quartiles of CVH‐HEI scores was 0.53 (95% confidence interval [CI]: 0.37, 0.77), and the association (HR: 0.57, 95% CI: 0.38, 0.84) did not materially change after adjusting for multiple covariates. The HR of all‐cause mortality per 10 points increase in CVH‐HEI scores was 0.85 (95% CI: 0.75, 0.96) in the multivariate models. Similar results were observed when we used the DASH diet pattern to calculate CVH scores; the results showed that the multivariate‐adjusted HR for the highest versus lowest quartiles of CVH‐DASH scores was 0.53 (95% CI: 0.36, 0.80) (Table 2).

**Table 2:** Adjusted HR and 95% CIs for risk of all‐cause death across CVH score quartiles in individuals with cardiovascular diseases.

	CVH, HR (95%CI)					
	Quartile1	Quartile2	Quartile3	Quartile4	Each 10 points increment	*p*_trend_
CVH‐HEI						
Case/person‐years	66/2023	51/2135	63/2086	49/2204		
Median (IQR)	41.3 (36.9, 44.4)	51.3 (48.8, 53.1)	59.4 (57.5, 61.2)	68.8 (66.3, 73.8)		
Age‐adjusted	1.00 (Reference)	0.64 (0.44, 0.93)	0.67 (0.47, 0.96)	0.53 (0.37, 0.77)	0.83 (0.74, 0.93)	.001
Multivariate‐adjusted^a^	1.00 (Reference)	0.65 (0.45, 0.95)	0.70 (0.49, 1.01)	0.57 (0.38, 0.84)	0.85 (0.75, 0.96)	.009
CVH‐DASH						
Case/person‐years	66/2003	55/2125	62/2137	46/2184		
Median (IQR)	41.3 (37.5, 44.4)	51.9 (49.4, 54.4)	60.00 (57.5, 61.9)	70.0 (66.89, 74.5)		
Age‐adjusted	1.00 (Reference)	0.62 (0.43, 0.89)	0.63 (0.44, 0.89)	0.49 (0.33, 0.71)	0.83 (0.74, 0.93)	<.001
Multivariate‐adjusted	1.00 (Reference)	0.65 (0.45, 0.94)	0.67 (0.46, 0.96)	0.53 (0.36, 0.80)	0.85 (0.76, 0.96)	.003

Abbreviations: CI, confidence interval; CVH: cardiovascular health; DASH: dietary approaches to stop hypertension; HEI: healthy eating index; HR: hazard ratio; IQR, interquartile range.

^a^ Multivariate‐adjusted model was adjusted for age, sex (men or women), family history of myocardial infarction and stroke (yes or no), waist circumference (quartiles), high sensitivity C‐reactive protein (≤3, >3 mg/L), estimated glomerular filtration rate (quartiles), alcohol consumption (never and past, current or missing), glucose‐lowering medications (yes or no), lipid‐lowing medications (yes or no), antihypertensive medications (yes or no), educational level (illiterate and primary, middle school, high school, and above), occupation (white collar, coal miner, or blue collar), monthly salary (≤1000, 1001–3000, >3000 RMB, or missing).

**Figure 1:**
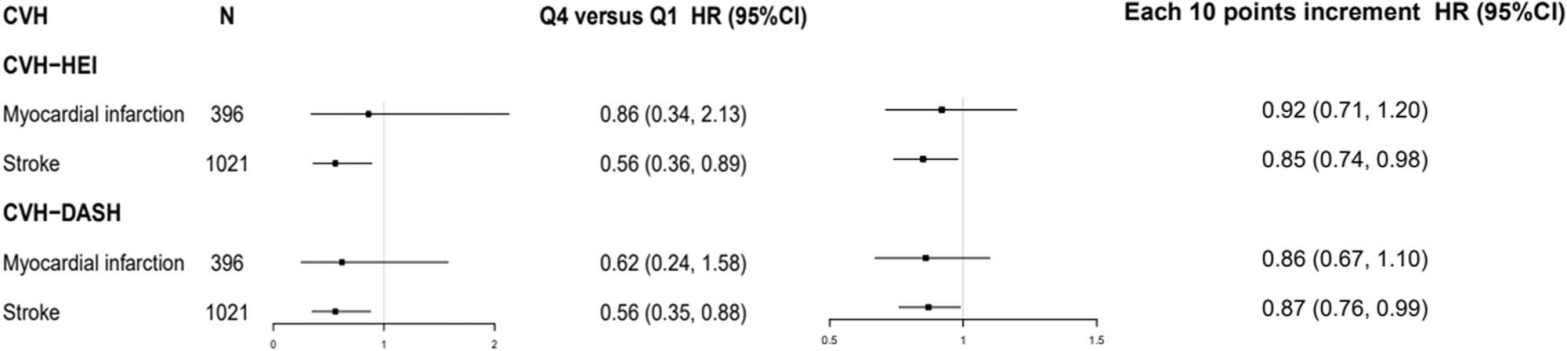
Adjusted HRs and 95% confidence intervals for risk of all‐cause mortality across CVH score quartiles according to the types of cardiovascular disease. Model was adjusted for age, sex (men or women), family history of myocardial infarction and stroke (yes or no), waist circumference (quartiles), high sensitivity C‐reactive protein (≤3, >3 mg/L), estimated glomerular filtration rate (quartiles), alcohol consumption (never and past, current or missing), glucose‐lowering medications (yes or no), lipid‐lowing medications (yes or no), antihypertensive medications (yes or no), educational level (illiterate and primary, middle school, high school, and above), occupation (white collar, coal miner, or blue collar), monthly salary (≤1000, 1001–3000, >3000 RMB, or missing). CVH, cardiovascular health; DASH: dietary approaches to stop hypertension; HEI, healthy eating index; HR, hazard ratio.

Significant results were observed only in participants with stroke (*n* = 1021) but not for MI (*n* = 396) when participants were stratified by CVD types (Figure 1).

**Figure 2:**
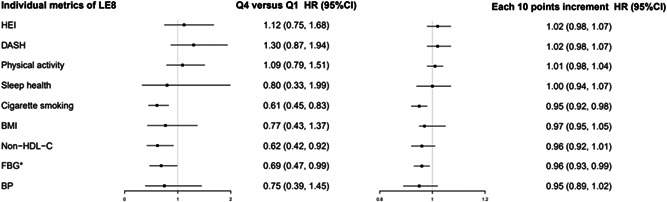
Adjusted HRs and 95% confidence intervals for risk of all‐cause mortality across individual metrics of cardiovascular health score *The HR value was the highest tertiles of FBG compared with the lowest tertiles. Model was adjusted for age, sex (men or women), family history of myocardial infarction and stroke (yes or no), waist circumference (quartiles), high sensitivity C‐reactive protein (≤3, >3 mg/L), estimated glomerular filtration rate (quartiles), alcohol consumption (never and past, current or missing), glucose‐lowering medications (yes or no), lipid‐lowing medications (yes or no), antihypertensive medications (yes or no), educational level (illiterate and primary, middle school, high school, and above), occupation (white collar, coal miner, or blue collar), monthly salary (≤1000, 1001–3000, >3000 RMB, or missing). BMI, body mass index; BP: blood pressure; FBG, fasting blood glucose; HDL‐C, high‐density lipoprotein cholesterol; HR, hazards ratio; LE8, life's essential 8.

We further analyzed the association between individual metrics of LE8 and all‐cause mortality (Figure 2). The results showed that higher cigarette smoking, non‐HDL‐C, and FBG scores were significantly associated with lower all‐cause mortality risks.

No significant interactions between the CVH scores and age, sex, and duration of CVD diagnosis were observed (*P*_interaction_ > 0.05 for all); thus, we did not conduct subgroup analyses further.

In sensitivity analyses, the association between CVH scores and all‐cause mortality risk was similar when excluding coal miners or mortality cases occurring during the first 2 years of follow‐up (Supporting Information: Figure S2).

## 4 DISCUSSION

In this community‐based study of 1391 participants diagnosed with CVD, we found a significant association between a higher CVH score assessed by the LE8 metrics and a lower risk of all‐cause mortality. Participants with the highest CVH scores compared with the lowest CVH scores had nearly 50% lower risk for all‐cause mortality during approximately 6 years of follow‐up.

Our findings are consistent with a previous study using “life's simple 7” (LS7) to assess CVH and observed that stroke survivors who met four or more health metrics had a 49% reduction in all‐cause mortality.^26^ LS7 is a CVH index proposed by the American Heart Association in 2010, including diet, PA, smoking, BMI, lipids, FBG, and BP.^27^ Although the original purpose of the CVH assessed by LE8 was to predict CVD risks in general populations,^6^ our study, together with the previous one based on LS7,^26^ suggests that LE8 could be of clinical significance for secondary prevention.

In the analyses of individual metrics, the higher smoking, lipids, and blood glucose scores were associated with a lower risk of all‐cause mortality, similar to previous studies. A study included 32 738 participants with stable coronary artery disease from 45 countries and found that compared with never‐smokers, both current and former smokers were associated with a higher risk of all‐cause death and cardiovascular death within 5 years.^4^ Another study showed that low adherence to statin therapy was associated with higher mortality risk in participants with atherosclerotic CVD.^5^ However, we did not observe significant associations of the other individual metrics, such as diet, PA, and sleep duration, with mortality, which was inconsistent with previous studies.^3^, ^28^ The distinct findings may be due to the sample size, participants' characteristics, and variable definition and division, which limit to detection of the small‐to‐modest effects of these factors on mortality in individuals with CVD. However, this also provides evidence to support the notion that using a comprehensive CVH indicator, such as LE8, may help us find a stable estimate of the CVH‐mortality relationship in individuals with CVD.

No significant interactions of CVH scores with age, sex, and duration of CVD were observed in this study. This could be due to relative sample size in each subgroup; for example, the proportion of women was only 10%, which may limit the statistical power. Nevertheless, the results of this study still provide important implications, suggesting that adopting healthy lifestyles and maintaining appropriate metabolic levels after a CVD diagnosis could reduce the risk of all‐cause mortality, regardless of these important determinants for mortality.

## 5 LIMITATIONS

Several limitations in this study should be noted. The CVH scores assessed by LE8 were only based on the 2014 survey. However, the results in previous studies also conducted in the Kailuan study showed that higher CVH scores were associated with a low risk of CVD in both baseline and long‐term changes.^29^, ^30^ Nevertheless, repeated assessment of the CVH scores is warranted for future study. As mentioned before, the sample size limited our subgroup analyses, including stroke types, age, and sex. Further, individuals with CVD who were excluded in the current analysis because of the lack of completed CVH data in this study were older, had higher levels of hs_CRP, and had a higher proportion of antihypertensive medication use, which were associated with a higher risk of mortality. Therefore, we might underestimate the association between CVH score and all‐cause mortality in individuals with CVD. Finally, the generalizability of results may be limited. We excluded participants who worked in coal mines, and the results were similar to the main findings. However, women were underrepresented in the studied population, and thus the results should be interpreted with caution in this context.

## 6 CONCLUSION

This study suggested that participants with high CVH scores had a nearly half lower risk of all‐cause mortality relative to those with poor CVH status. The findings have significant public health and clinical implication for emphasizing the importance of regular monitoring of lifestyle and metabolic factors in individuals with CVD. Further studies with larger sample sizes, longer follow‐up duration, and repeated assessment of the CVH scores are needed.

## AUTHOR CONTRIBUTIONS

Dr. Xiang Gao and Dr. Shouling Wu had full access to all of the data in the study and take responsibility for the integrity of the data and the accuracy of the data analysis. *Concept and design*: Shouling Wu and Xiang Gao. *Acquisition, analysis, or interpretation of data*: All authors. *Drafting of the manuscript*: Lili Huang. *Critical revision of the manuscript for important intellectual content*: Yan Zheng, Shouling Wu, and Xiang Gao. *Statistical analysis*: Lili Huang and Aitian Wang. *Obtained funding*: Zhijun Wu and Xiang Gao. *Supervision*: Xiang Gao.

## CONFLICT OF INTEREST STATEMENT

The authors declare no conflict of interest.

## Supporting information

Supporting information.

## Data Availability

The data are available from the corresponding authors upon reasonable request.
