# Supplementary material for: Life's essential 8 and risk of all‐cause mortality in individuals with cardiovascular diseases: A prospective community‐based study
Source: Clin Cardiol. 2023 Nov 23;47(2):e24119. doi: 10.1002/clc.24119 (PMC10823447; doi:10.1002/clc.24119)
Supplement: Supplementary file 1 — Supporting information. [file CLC-47-e24119-s001.docx]

**Table S1. Baseline characteristics of participants with and without cardiovascular health data**

|  | **With CVH data** | **Without CVH data** | ***P*** |
| --- | --- | --- | --- |
| N | 1391 | 2551 |  |
| Age, year | 62.6±9.30 | 67.8±9.82 | <0.001 |
| Men, % | 89.2 | 89.4 | 0.85 |
| Family history of CVD, % | 12.5 | 7.8 | <0.001 |
| Lipid-lowering medications, % | 4.3 | 2.9 | 0.02 |
| Antihypertensive medications, % | 36.4 | 42.5 | <0.001 |
| Glucose-lowering medications, % | 10.4 | 0.1 | 0.17 |
| Education level, % |  |  |  |
| Illiterate and Primary | 7.8 | 18.2 | <0.001 |
| Middle school | 77.6 | 63.7 |  |
| High school and above | 14.6 | 11.5 |  |
| Missing | - | 6.6 |  |
| Occupation, % |  |  |  |
| Coalminers | 21.0 | 27.9 | <0.001 |
| Other blue collars | 67.4 | 59.3 |  |
| White collars | 9.0 | 5.4 |  |
| Missing | 2.6 | 7.4 |  |
| Average income per month, % |  |  |  |
| ≤1000RMB | 42.0 | 20.8 | <0.001 |
| 1001-3000RMB | 48.6 | 44.6 |  |
| >3000RMB | 8.2 | 27.8 |  |
| Missing | 1.2 | 6.9 |  |
| Hs_CRP>3mg/L, % | 21.8 | 25.7 | 0.003 |
| WC, cm | 89.2±9.29 | 90.1±9.57 | 0.007 |
| eGFR, ml/min/1.73m^2^ | 90.4±20.2 | 81.1±37.0 | <0.001 |

CVH: cardiovascular health; CVD: Cardiovascular disease; Hs_CRP: high-sensitivity C-reactive protein; WC: waist circumference; eGFR: estimated glomerular filtration rate


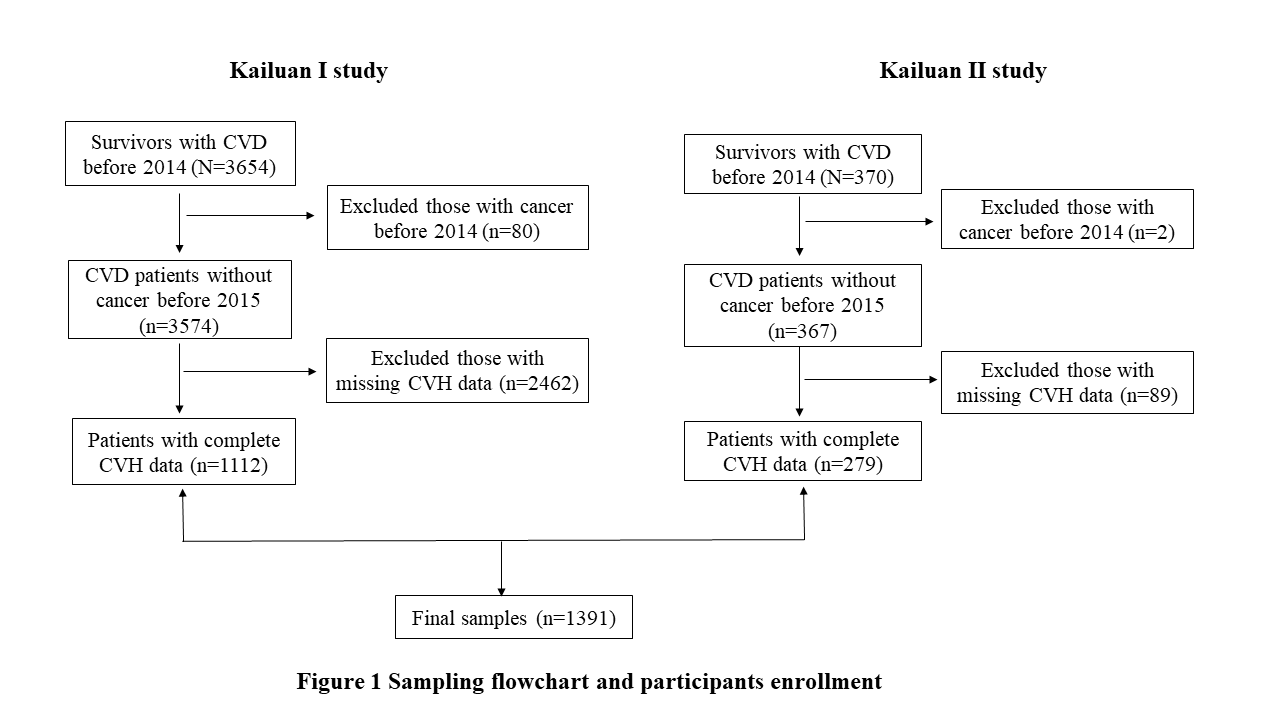


**Figure S1 Sampling flowchart and participants enrollment**


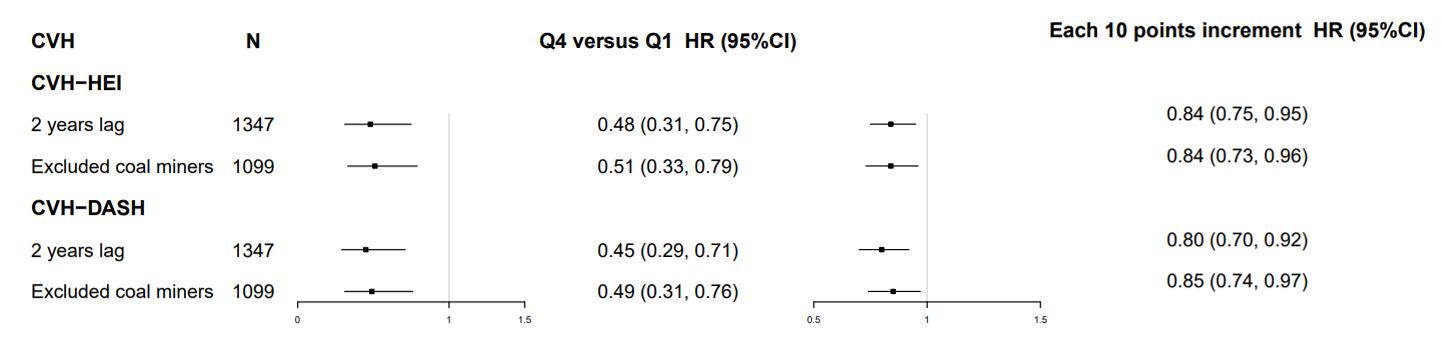


**Figure S2. Sensitive analyses for the association between cardiovascular health scores and all-cause mortality risk in individuals with cardiovascular diseases**

Model was adjusted for age, sex (men or women), family history of myocardial infarction and stroke (yes or no), waist circumference (quartiles), high sensitivity C-reactive protein (≤3mg/L, >3mg/L), estimated glomerular filtration rate (quartiles) , alcohol consumption (never and past, current or missing), glucose-lowering medications (yes or no), lipid-lowing medications (yes or no), antihypertensive medications (yes or no), educational level (illiterate and primary, middle school, high school and above), occupation (white collar, coal miner or blue collar), monthly salary (≤1000, 1001-3000, >3000RMB or missing).
